# Adaptive Mutations and Replacements of Virulence Traits in the *Escherichia coli* O104:H4 Outbreak Population

**DOI:** 10.1371/journal.pone.0063027

**Published:** 2013-05-10

**Authors:** Lionel Guy, Cecilia Jernberg, Jenny Arvén Norling, Sofie Ivarsson, Ingela Hedenström, Öjar Melefors, Ulrika Liljedahl, Lars Engstrand, Siv G. E. Andersson

**Affiliations:** 1 Department of Molecular Evolution, Cell and Molecular Biology, Science for Life Laboratory, Biomedical Centre, Uppsala University, Uppsala, Sweden; 2 Department of Preparedness, Swedish Institute for Communicable Disease Control, Solna, Sweden; 3 Department of Microbiology, Tumor- and Cell Biology, Karolinska Institutet, Stockholm, Sweden; 4 Department of Medical Sciences, Molecular Medicine, Uppsala University, Uppsala, Sweden; Cornell University, United States of America

## Abstract

The sequencing of highly virulent *Escherichia coli* O104:H4 strains isolated during the outbreak of bloody diarrhea and hemolytic uremic syndrome in Europe in 2011 revealed a genome that contained a Shiga toxin encoding prophage and a plasmid encoding enteroaggregative fimbriae. Here, we present the draft genome sequence of a strain isolated in Sweden from a patient who had travelled to Tunisia in 2010 (E112/10) and was found to differ from the outbreak strains by only 38 SNPs in non-repetitive regions, 16 of which were mapped to the branch to the outbreak strain. We identified putatively adaptive mutations in genes for transporters, outer surface proteins and enzymes involved in the metabolism of carbohydrates. A comparative analysis with other historical strains showed that E112/10 contained Shiga toxin prophage genes of the same genotype as the outbreak strain, while these genes have been replaced by a different genotype in two otherwise very closely related strains isolated in the Republic of Georgia in 2009. We also present the genome sequences of two enteroaggregative *E. coli* strains affiliated with phylogroup A (C43/90 and C48/93) that contain the *agg* genes for the AAF/I-type fimbriae characteristic of the outbreak population. Interestingly, C43/90 also contained a *tet/mer* antibiotic resistance island that was nearly identical in sequence to that of the outbreak strain, while the corresponding island in the Georgian strains was most similar to *E. coli* strains of other serotypes. We conclude that the pan-genome of the outbreak population is shared with strains of the A phylogroup and that its evolutionary history is littered with gene replacement events, including most recently independent acquisitions of antibiotic resistance genes in the outbreak strains and its nearest neighbors. The results are summarized in a refined evolutionary model for the emergence of the O104:H4 outbreak population.

## Introduction

Since the beginning of May 2011 to the beginning of July 2011, we have witnessed the largest outbreak of Shiga toxin-producing *Escherichia coli* (STEC) reported to date in Europe [1]. A case-control study of 26 infected subjects and 81 control subjects showed that bean sprout consumption explained 100% of the cases [2]. The outbreak resulted in more than 3,000 cases of STEC-diarrhea and more than 800 cases of hemolytic uremic syndrome (HUS), most of which occurred in Germany [1]. Whereas typical STEC outbreaks mostly affect children younger than 5 years, the majority of the cases in this outbreak were adults, with an overrepresentation of female patients [1]. Hemolytic uremic syndrome is most often caused by Shiga toxin producing enterohemorragic *E. coli* (EHEC) strains of serotype O157:H7 that belong to phylogroup E. Surprisingly, early investigations showed that the outbreak strain was of serotype O104:H4 and contained adherence properties similar to enteroaggregative *E. coli* (EAEC) strains of phylogroup B1 [3], [4], [5].

The first genome of an outbreak strain to be sequenced was isolated from a 16-year-old girl (TY2482) [6]. The assembled sequence was most similar to the EAEC strain 55989, isolated from an HIV-positive patient in Central Africa in year 2002 [6], [7]. A comparison of the two strains identified segments with phage genes solely present in the outbreak strain. One of these contained the Shiga toxin genes, which were identical to the Shiga toxin genes of the *E. coli* O157:H7 strain except for a single nucleotide polymorphism. Also identified were three plasmids, one of 76 kb (pEAEC) that encodes the aggregative adherence fimbriae (AAF/I), another of 88 kb (pESBL) that encodes extended-spectrum beta-lactamase CTX-M-15 and a third small plasmid of 1.5 kb.

A second study sequenced the genome of the German outbreak strain LB226692 and a historical STEC O104:H4 strain isolated in 2001 (01-09591) [8]. Both genomes contained the Shiga toxin genes, but differed in their plasmid contents. The largest plasmid in 01-09591 was 95 kb and highly similar to plasmid pSERB1 from the EAEC strain C1096, which belongs to the IncI family of plasmids. Another plasmid of 75 kb encoded the aggregative adherence fimbriae, but these were of type AAF/III rather than of the AAF/I type, as in the outbreak strain.

A third study gathered genomic data from strain C227-11 isolated from a 64-year-old woman from Germany who was hospitalized in Copenhagen [9]. Additionally, the genomes of six African O104:H4 enteroaggregative *E. coli* isolates and five other reference enteroaggregative strains were sequenced [9]. However, the relationships of the African isolates to the outbreak strains and the O104:H4 strains 55989 and 01-09591 could not be determined due to the high error rate of the PacBio instrument used for the sequencing of these strains. Most recently, the first complete genome sequence of isolate 2011C-3493 obtained from a US patient who had been travelling to Germany during the outbreak was presented along with genome data from two historical strains isolated in the Republic of Georgia in 2009 (2009EL-2050 and 2009EL-2071) [10]. A comparison of these strains revealed differences in antibiotic resistance genes as well as in prophage sequences and plasmids despite an otherwise extremely close evolutionary relationship.

The model for the evolution of the outbreak strains has gradually been refined as more closely related historical strains have been sequenced. By now, most of the inferred virulence traits have also been identified in one or more of the historical strains, suggesting that they were acquired already prior to the emergence of the outbreak. For example, the pEAEC plasmid encoding the enteroaggregative phenotype and the prophage associated Shiga toxin genes (*stxA*) have been identified in several of the historical strains. However, the genotypes are not always the same, indicating one or more replacement events. For example, the Shiga toxin phage genotypes were found to be different in the outbreak and the closely related historical strains from Georgia [10]. This was thought to reflect a recent gene transfer events into the outbreak strain, which was suggested to explain its high apparent pathogenicity [10].

In this study, we infer adaptive mutations in the outbreak genome through comparisons with a closely related historical strain from Tunisia. We use phylogenetic analyses to identify the donors of virulence features acquired throughout the evolution of the outbreak strain and show that the postulated replacement of the Shiga toxin genes occurred in the 2009 historical strains rather than in the outbreak strain.

## Results

### Whole-genome SNP Phylogeny

We have sequenced the genome of three *E. coli* strains isolated prior to the outbreak and tested positive in enteroaggregative assays like the outbreak strains. Strain E112/10 was of the same serotype O104:H4 as the outbreak strains, but was isolated a year earlier, in 2010, from a Swede who had been travelling in Tunisia. Strain C43/90 was classified as serotype O127:H27 and was isolated from a one-year old patient in 1990 who got infected while traveling in Turkey, while strain C48/93 was isolated in 1993 and was classified as serotype ONT: H33. The draft sequences of these genomes were compared to all complete *E. coli* and *Salmonella* genomes available at NCBI, all of the historical strains that were most closely related to the outbreak and available as draft sequences (Table S1) as well as a few outbreak strains [9], [11].

**Figure 1:**
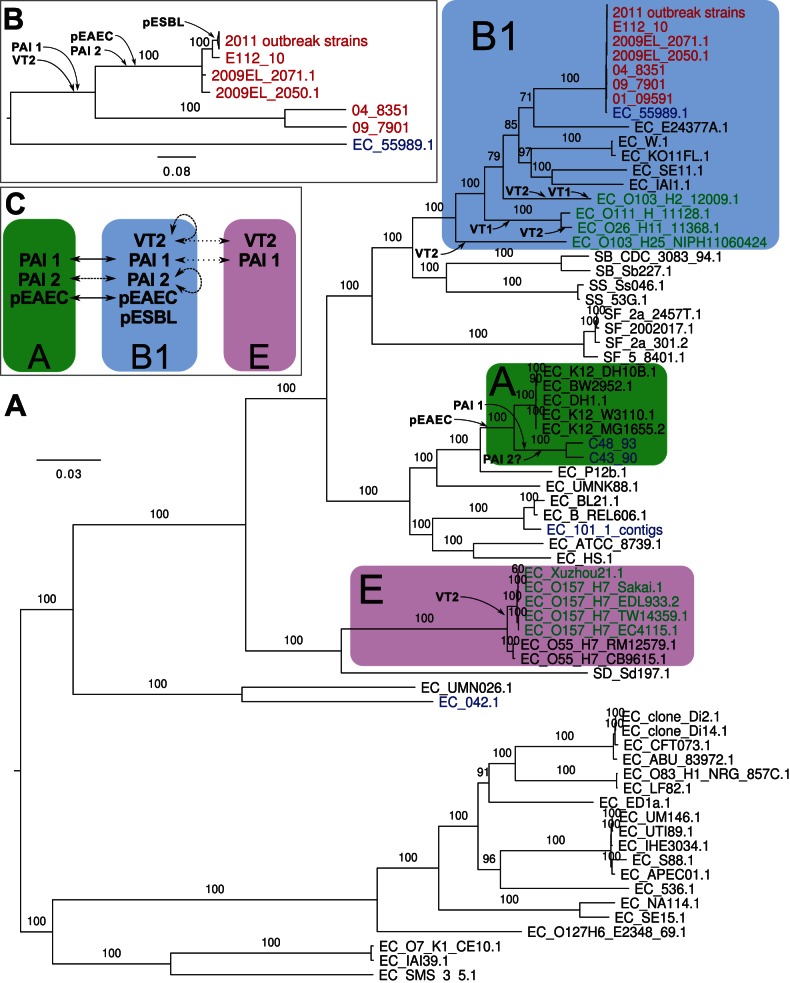
Phylogeny of *E.coli* and *Salmonella* genomes. Maximum-likelihood phylogenies (**A**) of all *E. coli* and *Salmonella* strains for which genome data is available and (**B**) of selected O104:H4 strains and their closest non-O104:H4 strain, 55989. Font coloring, red: O104:H4 strains; blue: EAEC of non-O104:H4 serotype; green: EHEC strains. Phylogroups B1, A and E [7] are shown with blue, green and violet boxes, respectively. The probable insertions of prophages, genomic islands and plasmids are indicated. Bootstrap support higher than 70 (out of 100) is shown. To improve readability of the tree in panel A, bootstraps are not shown in the clade that is also depicted in panel B. (**C**) Inferred lateral transfers of virulence factors between phylogroups A, B1 and E.

We first inferred a whole-genome phylogeny of these strains based on an alignment of reads, contigs or whole-genome sequences mapped onto the non-repetitive parts of the complete, closed genome sequence of 2011C-3493, used in this study as the German outbreak reference strain. The phylogeny showed that the 2011 outbreak strains clustered with *E. coli* strains of the B1 phylogroup (Figure 1A). The Tunisian 2010 strain, E112/10, diverged immediately prior to the 2011 outbreak strains, after the two strains 2009EL-2050 and 2009EL-2071, isolated in Georgia in 2009 (100% bootstrap support; Figure 1B). This is interesting since the Georgian strains have until now been considered the most closely related strains to the outbreak strains.

The other two enteroaggregative strains sequenced as part of this study, C43/90 and C48/93, clustered with commensal *E. coli* K12 strains of the A phylogroup (Figure 1A). The historical strain C35-10 was also contained within this clade with 100% bootstrap support in a SNP phylogeny in which TY2482 was used as the reference (Figure S1). This was surprising since it was previously classified into the O104:H4 group [9]. However, a re-analysis of the alignment of C35-10 reads to TY2482 suggested that many SNPs were not called in the initial analysis. Although our analysis excludes a placement near to the outbreak strains, the low quality of the PacBio reads used for the sequencing of strains C35-10, JM221 and 17_2 makes it difficult to assign their correct placements within the A-group, and they might be more closely related to C48/93 and C43/90 than indicated here (Figure S1).

### Adaptive Nucleotide Substitutions in the Outbreak Strain

Strain E112/10 differed from 2011C-3493 by 42 SNPs in single copy genes, as inferred from a filtered SNP matrix in which all phage sequences had been manually removed. Of the 42 SNPs, 4 were specific to 2011C-3493, whereas 38 were shared by all outbreak strains examined thus far [11]. We mapped these 38 SNPs onto either 2011C-3493 or E112/10 and determined which of the two bases was present in the historical strains 2009EL-2050, 2009EL-2071, 04-8351, 09-7901 and 55898. The base that was shared by the historical strains was deemed as being the ancestral state, and the mutation was thus attributed to the branch leading to strain having the other base. Using this approach, we were able to unambiguously associate 16 mutations with the branch leading to the outbreak strains and another 22 to the branch leading to E112/10 (Table 1).

**Table 1:** SNPs between E112/10 and the *E. coli* outbreak German isolates.

Position in 2011C-3493	Position in TY2482, rotated	SNP	Loc^a^	N^b^	AA^c^	Locus tag	Gene	Annotation	Strain
374280	4079071	C->A	CDS	N	D->E	O3K_01800	*ugpB*	glycerol-3-phosphate transporter periplasmic binding protein	E112/10
380310	4073041	G->A	CDS	S	*–*	O3K_01830	*ggt*	gamma-glutamyltranspeptidase	E112/10
680072	3773277	A->G	CDS	N	F->S	O3K_03335	*–*	hypothetical protein	E112/10
1135122	3317489	C->T	CDS	N	A->T	O3K_05510	*sdaB*	L-serine deaminase II	E112/10
1348726	3103699	A->T	CDS	S	-	O3K_06535	*nadB*	quinolinate synthase, L-aspartate oxidase	E112/10
1413160	3039265	C->T	CDS	N	P->L	O3K_06820	*pbpC*	penicillin-binding protein 1C	E112/10
1649869	2802473	C->A	CDS	N	R->L	O3K_07975	*–*	NAD dependent epimerase/dehydratase family protein	E112/10
2087027	2365134	G->A	CDS	N	S->F	O3K_09945	*fliF*	flagellar MS-ring protein	E112/10
2212137	2240010	C->T	CDS	S	*–*	O3K_10675	*zwf*	glucose-6-phosphate 1-dehydrogenase	E112/10
2413202	2038897	C->T	CDS	N	G->S	O3K_11765	*–*	acetyl-CoA: acetoacetyl-CoA transferase subunit alpha/beta	E112/10
2461442	1990657	A->T	CDS	N	Y->N	O3K_11980	*nemA*	N-ethylmaleimide reductase	E112/10
3235407	1215568	C->T	CDS	N	L->F	O3K_15870	*pgaB*	outer membrane N-deacetylase	E112/10
3239060	1211915	T->C	ig	*–*	*–*	*–*	*–*	*–*	E112/10
3288859	1161972	A->G	CDS	S	*–*	O3K_16115	*–*	hypothetical protein	E112/10
3313293	1137538	G->A	CDS	N	V->I	O3K_16290	*–*	regulatory protein	E112/10
3520805	930095	T->G	CDS	N	L->V	O3K_17215	*moeB*	molybdopterin biosynthesis protein MoeB	E112/10
4130999	319547	C->T	ig	*–*	*–*	*–*	*–*	*–*	E112/10
4157098	293448	C->T	ig	*–*	*–*	*–*	*–*	*–*	E112/10
4373593	76954	A->G	CDS	N	E->G	O3K_21210	*sgrR*	transcriptional regulator SgrR	E112/10
4518367	5211133	G->A	CDS	N	A->T	O3K_21840	*–*	inner membrane protein	E112/10
4667748	5061192	G->A	CDS	N	V->I	O3K_22600	*–*	putative cell envelope opacity-associated protein	E112/10
5176186	4552535	C->T	CDS	N	R->H	O3K_24910	*rffA*	TDP-4-oxo-6-deoxy-D-glucose transaminase	E112/10
174510	4279463	A->C	CDS	N	N->H	O3K_00895	*selB*	selenocysteinyl-tRNA-specific translation factor	Outbreak
605339	3848010	T->C	CDS	N	I->M	O3K_02960	*-*	putative calcium/sodium:proton antiporter	Outbreak
624647	3828702	A->G	CDS	N	T->A	O3K_03070	*–*	hydrolase, inner membrane	Outbreak
1438448	3013977	A->C	CDS	N	E->D	O3K_06930	*upp*	uracil phosphoribosyltransferase	Outbreak
1584996	2867346	A->G	CDS	N	R->G	O3K_07665	*–*	portal protein p19^d^	Outbreak
1757038	2695256	A->G	CDS	N	H->R	O3K_08425	*napG*	quinol dehydrogenase periplasmic component	Outbreak
1883285	2569009	T->C	CDS	S	*–*	O3K_08990	*gatC*	PTS system galactitol-specific transporter subunit IIC	Outbreak
1951216	2500990	G->A	CDS	S	-	O3K_09260	*-*	Wzx	Outbreak
2288948	2163199	G->A	ig	*–*	*–*	*–*	*–*	*–*	Outbreak
2668508	1783405	A->G	CDS	N	I->V	O3K_13035	*gadC*	glutamate/gamma-aminobutyrate antiporter	Outbreak
2859175	1592738	G->A	CDS	N	R->Q	O3K_13895	*–*	oxidoreductase	Outbreak
3956440	494106	G->A	CDS	N	T->I	O3K_19295	*lon*	DNA-binding ATP-dependent protease La	Outbreak
4039977	410569	A->G	CDS	N	W->R	O3K_19710	*mhpT*	putative 3-hydroxyphenylpropionic transporter MhpT	Outbreak
4078362	372184	T->G	CDS	N	*->Y	*–*	*–*	putative deaminase/amidohydrolase	Outbreak
4518246	5211254	T->G	ig	*–*	*–*	*–*	*–*	*–*	Outbreak
5053944	4674752	A->G	CDS	N	E->G	O3K_24345	*rhaB*	rhamnulokinase	Outbreak

a Loc = Location of the mutation. CDS: inside a coding sequence; ig: intergenic.

b N = Type of nucleotide substitution. N: nonsynonymous substitution; S: synonymous substitution; -: not applicable.

c AA = Amino acid change.

d SNP not present in 2009EL-2050.

The overall ratio of mutations in coding versus noncoding regions for these mutations was 6.6, which is close to an estimated ratio of 7.0 (Table 2). In genic regions, nonsynonymous substitutions were 3.75-fold more frequent than synonymous substitutions in the E112/10 genome, and as much as 6-fold more common in the outbreak genome. The latter ratio is almost twice as high as an expected value of 3.25 if all mutations are neutral. Transitions dominated in both strains, but the spectrum of mutations differed: 17 out of 22 substitutions in E112/10 lineage were from GC -> AT (77.3%), whereas 12 of the 16 substitutions in the outbreak genome were from AT -> GC (75%), indicative of positive selection.

**Table 2:** Summary of the number of SNPs between E112/10 and the *E. coli* outbreak German isolates.

	E112/10	*%*	Outbreak	*%*	Total	*%*
SNPs	22	*100*	16	*100*	38	*100*
Transitions	5	*22.7*	4	*25*	9	*23.7*
Transversions	17	*77.3*	12	*75*	29	*76.7*
AT->GC	5	*22.7*	12	*75*	17	*44.7*
GC->AT	17	*77.3*	4	*25*	21	*55.3*
Intergenic	3	*13.6*	2	*12.5*	5	*13.2*
Coding	19	*86.4*	14	*87.5*	33	*86.8*
Synonymous^a^	4	*21.1*	2	*14.3*	6	*18.2*
Non-synonymous^a^	15	*78.9*	12	*85.7*	27	*81.8*

a Percentage of synonymous and non-synonymous mutations are given with respect to mutations in coding sequences.

The genes affected by these mutations encode proteins involved in functional categories such as metabolism of carbohydrates, hydrolases, transporters and outer surface proteins (Table 2). More specifically, we identified putatively adaptive changes in the outbreak population in a gene for a transporter required during growth at low pH (*gadC*) [12], in an enzyme expressed during anaerobic conditions (*napG*) [13], and in a protein homologous to an enzyme involved in the hydrolysis of amide bonds in antibiotics such as β-lactamase [14]. Another nonsynonymous substitution was identified in the *mhpT* operon required for the catabolism of aromatic compounds such as phenylpropanoids that are breakdown products of lignin in plants and thought to be a common product in both human and animal diets [15].

Nonsynonymous substitutions mapped to the E112/10 genome were identified in genes coding for surface proteins, including target molecules for antibiotics and carbohydrate transporters. One such substitution was in the *pgaB* gene required for export of the polysaccharide PGA, which serves as an adhesin in biofilm formation involved in immunological defense [16]. Another substitution was in the *rffA* gene, which code for a molecule in the outer membrane glycolipid ECA [17]. A third unique mutation was identified in the *pbpC* gene, which is involved in peptidoglycan biosynthesis [18]. Upon binding of β-lactam antibiotics to the *pbpC* gene product cell wall biosynthesis is blocked [18]. Hence, mutations in this gene might confer a resistance phenotype. Two mutations were identified in genes involved in response to glucose concentrations; one was the *ugpB* gene, which codes for glycerol-3-phosphate transporter involved in the uptake of glycerol under starvation conditions [19]. Another was in the *sgrR* gene, which codes for a transcriptional regulator that activates transcription of the small RNA SgrS [20]. This regulatory RNA represses translation of mRNAs encoding sugar transporters under stress conditions [20].

### Acquisition and Replacement of Virulence Traits

**Figure 2:**
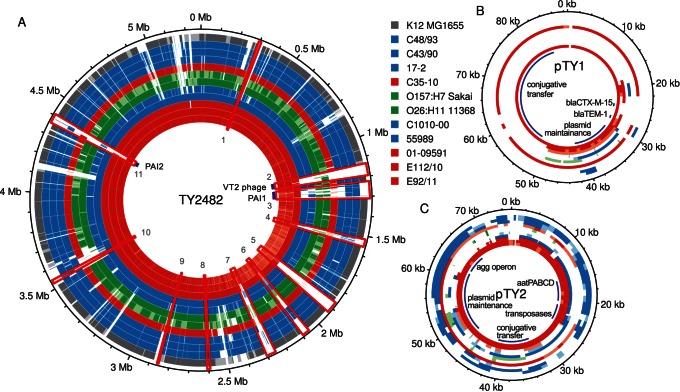
Comparison of the genomes and plasmids of *E.coli* strains. Comparisons of (**A**) chromosomes and (**B–C**) plasmids pTY1 and pTY2. Each circle corresponds to the alignment of one strain relative to TY2482. Numbering on the outside corresponds to the position on the genome of TY2482 after rotation to put the start of the sequence at the same position as in 55989 and K12. The chromosomal regions of difference between 55989 and TY2482 (Dataset S1) are shown in red boxes and numbered. For strains for which a full genome is available, regions are shown in white, pale or dark color if the identity between the regions is, respectively, lower than 95%, between 95% and 98%, and over 98%. For strains for which raw reads are available, regions are shown in white if the coverage in the alignment to TY2482 is lower than a standard deviation of the coverage distribution, in pale color if between this and the median coverage minus one standard deviation, and in dark color if above.

To identify putative replacement events as well as to trace the origin of genes putatively coding for virulence features of presumed importance for the outbreak, we examined the presence-absence patterns of the pEAEC plasmid coding for the enteroaggragative phenotype, the Shiga toxin coding prophage and a genomic island coding for antibiotic resistance phenotypes in the outbreak population and the historical strains (Figure 2, Dataset S1). However, given the dynamic nature of genomic islands and prophages, shared presence may not necessarily indicate shared ancestry. To decipher the evolutionary history and identify putative recombination events, we also performed phylogenetic analyses of these sequences.

#### Exchange of plasmids for enteroaggregative fimbriae

**Figure 3:**
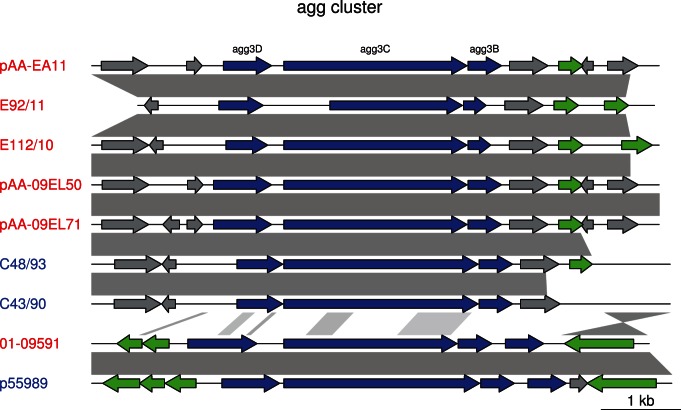
Comparison of the *agg* genes in the outbreak strains and in other EAEC strains. Blast hits between the plasmids are shown in grey. *agg* genes are shown by blue arrows, mobile genes (transposases, transposons and IS elements) are in green, and other genes in grey. The shorter *agg3C* gene in E92/11 is probably due to a sequencing error. The scale is indicated in the lower right corner.

The major difference between the outbreak strains and the historical strains resides in the plasmid pool. The pEAEC plasmid carrying the *agg* genes for the enteroaggregative phenotype is present in all historical strains, although the *agg* genes for AAF/III fimbriae in 01-09591 and 55989 have been replaced by *agg* genes for the AAF/I fimbriae prior to the diversification of the Georgian strains from the outbreak strains [10]. We identified the pEAEC plasmid carrying the *agg* genes for the AAF/I fimbriae also in E112/10, consistent with its clustering with the outbreak strains. Plasmids carrying the *agg* genes for the AAF/I fimbrae were also identified in the A-group strains C35-10, C43/90 and C48/93 (Figures 2 and 3), indicating access to a pool of plasmids that is shared with the outbreak population. The maintenance of the pEAEC plasmid throughout the evolution of the outbreak strains suggests that it evolves under strong selective constraints.

**Figure 4:**
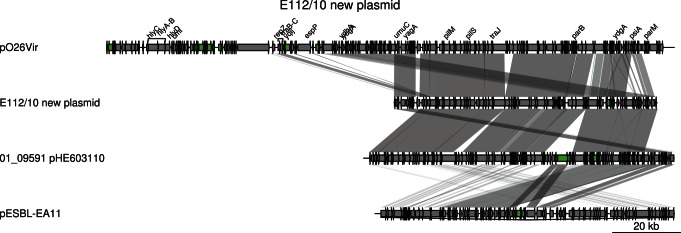
Comparison of the second large putative plasmid found in E112/10 with related plasmids. Comparisons are shown as in Figure 3. The two last plasmids (pHE603110 and pTY1) are shown in reverse orientation. Genes are shown by grey arrows, except for mobile genes (transposases, transposons and IS elements) (green). The scale is indicated in the lower right corner.

Additionally, each strain contained its own unique plasmid. In E112/10, we identified four contigs, totaling 79 kb, which are likely to form a second plasmid. These four contigs showed 99.4% sequence identity at nucleotide level to the IncI-type plasmid pHE603110, previously described in the historical O104:H4 strain 01-09591 [8]. However, a short cluster of genes in pHE603110 putatively conferring resistances to streptomycin, aminoglycoside/hydroxyurea and β-lactamase could not be identified in the E112/10 putative plasmid (Figure 4). The rest of the plasmid pHE603110 covered 100% of the four contigs in E112/10, indicating that the plasmids are of similar sizes although it cannot be excluded that the putative plasmid identified here is even longer. A novel plasmid of the IncF-type was detected in strain 2009EL-2050 [10], but there was no similarity between this plasmid and the plasmids identified in E112/10 and 01-09591. The pESBL plasmid that encodes the CTX-M-15 cephalosporinase (also called pTY1) in the outbreak strain could not be identified in E112/10 or any other historical isolate, although a shorter segment of about 20 kb in the pHE603110-like plasmid showed 96% sequence identity with pESBL (Figure 4).

#### Frequent recombination of genomic island genes for adhesins and tellurium resistance

**Figure 5:**
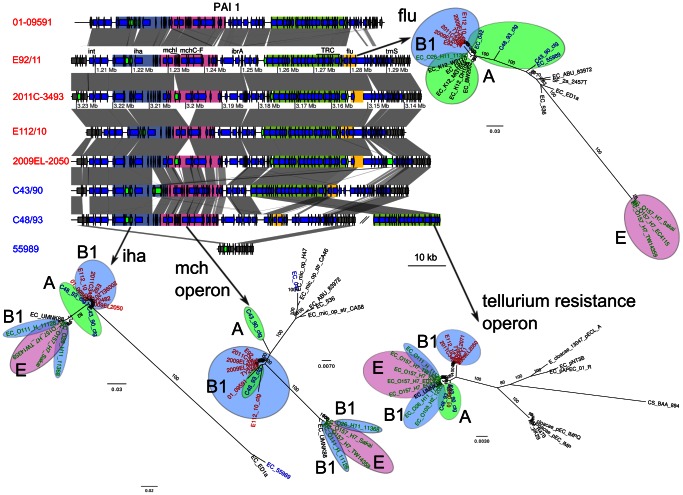
Comparison of the gene order structures of pathogenicity island 1 (PAI1). Genes located inside PAI1 are shown as blue arrows. Genes shown as green arrows are mobile genes (transposases, transposons and IS sequences). Four regions are defined with colors, and contain the *iha* gene (blue-grey), the microcin operon (pink), the tellurium resistance cluster (green) and the *flu* genes (yellow). Comparisons are shown as in Figure 3. The maximum-likelihood phylogenies of these regions are represented next to the gene map. Colors represent different subsets of strains, as in Figure 1.

The analyses also revealed similarities in chromosomal regions between the outbreak strains and C43/90 and C48/93, providing additional examples of gene exchange with A-group affiliated strains. One of these regions contained the *iha, mch, trc* and *flu* gene clusters coding for adhesins, microcins (small bacteriocins) and tellurium resistance (Figure 5). This region, here referred to as PAI1, is flanked by an integrase at its 5′ end and a tRNA at its 3′ end, and was identified in all historical strains except 55989, which only contained a highly divergent *iha* gene cluster. The phylogeny inferred from the *iha* gene cluster confirmed a close relationship of C43/90 and C48/93 with the outbreak strains, while the extent of sequence similarity was more variable for the other gene clusters in this region. For example, C48/93 clustered with the O104:H4 strains in the *mch* phylogeny while C43/90 was slightly more divergent. The *trc* gene region on the other hand indicated a cluster of C43/90 and C48/30 with the 2001 pre-outbreak strain 01-09591, while the other O104:H4 strains clustered elsewhere. Finally, all O104:H4 strains clustered in the *flu* gene phylogeny to the exclusion of the A-group strains C43/90 and C48/93. The many contradictory evolutionary signals in this region indicate multiple recombination events.

#### Independent acquisition of antibiotic resistance in the outbreak and the georgian strains

**Figure 6:**
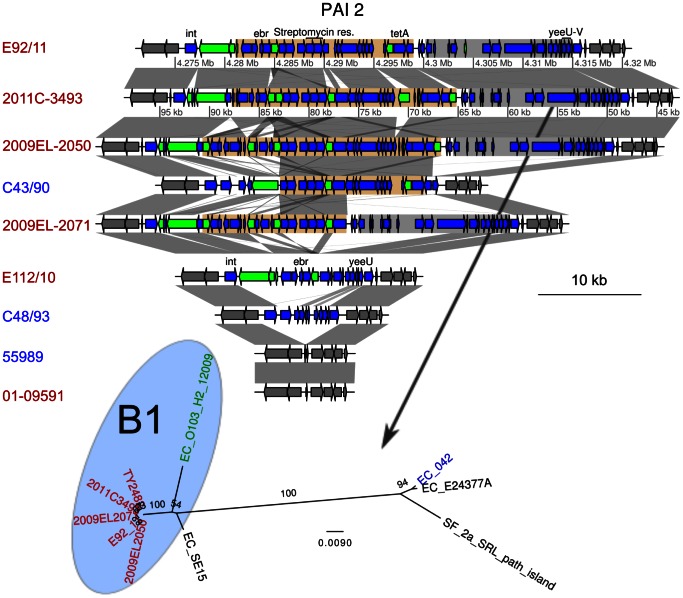
Comparison of the gene order structures of pathogenicity island 2 (PAI2). Genes located inside PAI2 are shown as blue arrows. Genes shown as green arrows are mobile genes (transposases, transposons and IS sequences). Two regions are defined with colors, and contain an antibiotic resistance cluster (light brown) and the 3′ region of the PAI (grey). The location of the integrase (int), ethidium bromide resistance gene (ebr), the streptomycin resistance genes, the tetramycin resistance gene (tetA) and the toxin-antitoxin genes *yeeU-yeeV* are shown. Comparisons are shown as in Figure 3. The maximum-likelihood phylogenies of the second regions is represented next to the gene map. The phylogeny of the first region was not inferred, due to too many rearrangements in this region. Colors represent different subsets of strains, as in Figure 1.

Another region contains multiple genes for antibiotic resistances, among which resistance to streptomycin, to tetracyclin and to ethidium bromide (Figure 6), as well as a *yeeV/yeeU* toxin/antitoxin system. This region, referred to here as PAI2, is flanked by a tRNA and an integrase at its 5′ end, and is completely missing in 55989. The antibiotic resistance cluster in PAI2, which is about 23 kb, was identified in 2009EL-2050, although the first 5 kb containing the ethidium bromide resistance were inverted compared to the outbreak strain 2011C-3493. The same inversion was observed in 2009EL-2071, but this strain is further missing the 10 last kb of the segment containing the *tetA* gene. We mapped the 454 single reads of the genomes sequenced here onto the fully sequenced genomes to examine gene presence/absence patterns in PAI2. Interestingly, a single contig in C43/90 harbored an exact copy of the last 13 kb of the cluster, including the streptomycin and the tetracyclin resistance genes. Most sequences in this region, including the antibiotic resistance genes, were absent from E112/10.

#### Replacement of the shiga toxin prophage in the georgian strains

**Figure 7:**
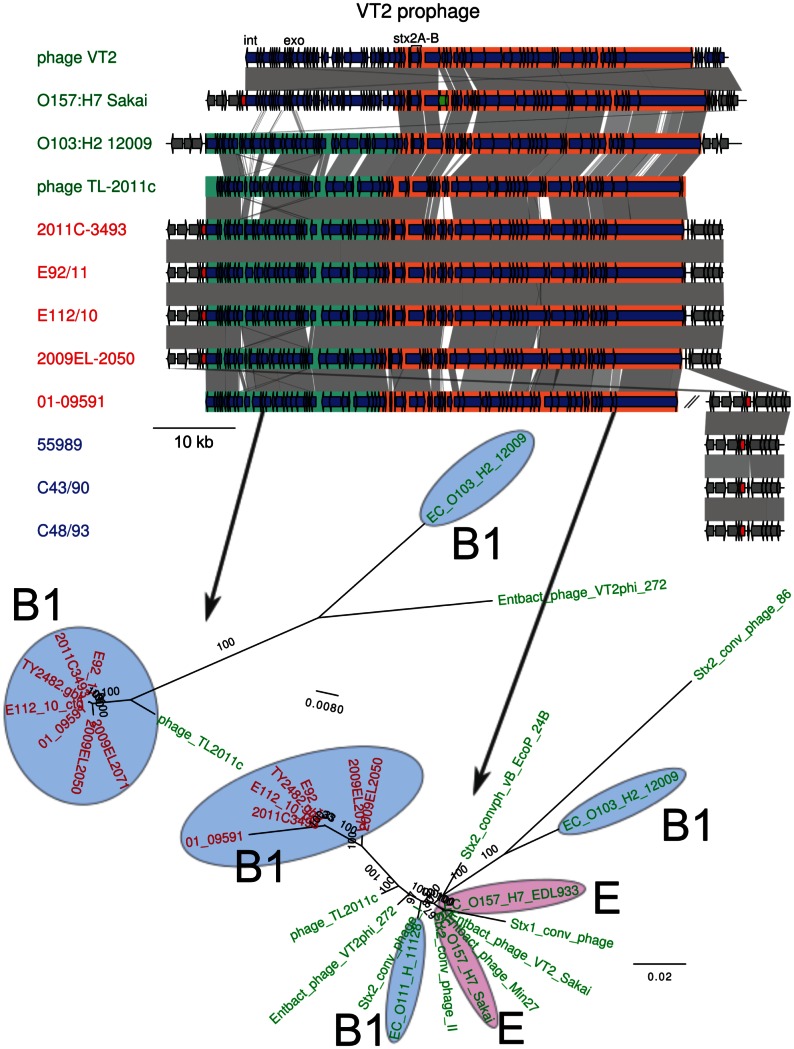
Comparison of the gene order structures of the VT2 phages. Genes located inside the prophage are shown as blue arrows. A transposase in O157:H7 Sakai is indicated in green. Two sub-regions have been defined and are shown in green and orange. The gene into which the insertion site is located, *wrbA*, is indicated in red. The location of the integrase (int), exonuclease (exo) and the Shiga toxin genes (stx2A, stx2B) are shown. For 01-09591, a contig containing *wrbA* is shown next to the contigs containing the prophage. Comparisons are shown as in Figure 3. The maximum-likelihood phylogeny of two sub-regions is shown below the map. Colors represent different subsets of strains, as in Figure 1.

The order of genes in the Shiga toxin prophage region (Figure 7) was similar in the outbreak strain and the historical strains and a phylogeny inferred from genes in this region distinguished the O104:H4 clade from all other strains, with phage TL2011c derived from a B1 strain of serotype O103:H25 representing the closest relative [21]. The phylogenies inferred from two sequence alignments showed a strong clustering of outbreak genomes with the historical strains E112/10 and 01-09591, to the exclusion of the 2009 strains from Georgia. This suggests that a replacement event occurred in the 2009 historical strains, rather than on the branch leading to the outbreak strains as suggested previously [10].

## Discussion

Strain E112/10, acquired by a Swede travelling in Tunisia a year prior to the outbreak is unique among the historical strains analyzed to date in that it differs by only 38 SNPs from the outbreak population. Our analyses concur with previous studies of historical strains, suggesting that multiple lineages that are closely related to the outbreak strain are circulating worldwide. Using the genome data from E112/10 and other historical strains enabled us to identify the most recent alterations in the genomes that preceded the outbreak in much greater detail than was possible previously (Figure 1C).

Firstly, we traced several unique substitutions to the branch of the outbreak population. Although the 38 SNPs were evenly distributed between the two lineages, the substitution patterns differed. The Tunisian strain displayed a 3-fold higher frequency of GC -> TA changes, consistent with a strong AT-mutation bias in *E. coli* and most other bacterial genomes [22]. In contrast, the outbreak strain featured a 3.5-fold higher frequency of AT -> GC changes and a frequency of nonsynonymous substitutions 4.5 fold higher that the frequency of synonymous substitutions. We hypothesize that some of the identified nucleotide changes leading to the outbreak strains were driven by positive selection for new variants with a higher fitness. Factors such as the pH and the availability of carbohydrates and other dietary compounds in the gut may well be specific to local host populations or individuals, enabling further fitness optimization.

The evolutionary model for the origin of the outbreak strain has been resolved in increasing detail as more closely related genomes have been sequenced [8], [10], [23]. The key events were the acquisition of the Shiga toxin prophage, the genomic islands and the plasmids for aggregative fimbriae and antibiotic resistances. Our results are largely consistent with the earlier models, but suggest that the current outbreak isolates have not acquired a new variant of the Shiga toxin prophage, as recently suggested [10]. Moreover, the absence of the antibiotic resistance plasmid pESBL in strain E112/90 strengthens the hypothesis that the acquisition of this plasmid is one of the most recent events in the evolutionary history of the outbreak strains.

Although all studies performed to date indicate similarities between the VT2 prophage carrying the Shiga toxin genes in animal- and human-adapted *E. coli* strains, there is no evidence indicating that the outbreak was caused by a Shiga toxin-producing strain adapted to cattle that was accidentally transmitted to humans. Rather, the genomic backbone of the outbreak strain – and thus the large majority of the genes – is related to *E. coli* strains that are also human-adapted. The clinical manifestations attributed to the Shiga toxin is therefore best explained by gene transfer of the Shiga toxin genes from the animal to the human adapted strains. Several of the historical strains contained nearly identical Shiga toxin-carrying prophages, suggesting that the toxin genes were transferred into the O104:H4 population long before the outbreak. Although the date of acquisition cannot be reliably determined, the human adapted O104:H4 population is likely to have harbored the VT2 phage for at least a decade, between 2001 (date of isolation of strain 01-09591) and the outbreak in 2011.

Phage genomes are normally highly mosaic in nature, and the Shiga toxin phage population consists of many different phage variants that are inducible by antibiotics to different extents and produce the toxin at different levels. It was recently proposed that two separate phage acquisition events occurred in the outbreak strain and the closely related Georgian strains [10] and that this may explain the higher pathogenicity of the outbreak strain. However, our study has shown that E112/10 and 01-09551 strains contain the same genotype as the outbreak strain, and that it is the Georgian strains that have acquired a novel genotype.

Finally, we have demonstrated recent transfer of virulence genes for aggregative fimbriae, adhesins and antibiotic resistances between the outbreak strain and strains that are otherwise affiliated with commensal E. coli strains of phylogroup A. The expression of enteroaggregative fimbriae is required for biofilm formation and adherence to the epithelium, and is thus considered another important virulence trait. The pEAEC plasmid carrying the genes for the enteroaggregative fimbriae was also acquired early in the history of the outbreak strain, but modified through subsequent replacement events. We have shown here that the agg genes for the AAF/I type of fimbriae located on the pEAEC plasmid as well as adhesins located in one of the variable genomic islands are present in the C43/90 and C48/93. Likewise, our study of sequences in the tet/mer islands for antibiotic resistances show evidence for recent replacement events, with the outbreak strain being identical to C43/90 in the terminal part of the island, whereas one of the two Georgian strains display a deletion of a large part of the same region. Of the inferred gene transfer events it is the acquisition of a novel antibiotic resistance plasmid and a gene cassette for antibiotic resistances that distinguishes the outbreak strain from the related historical strains.

Thus, all evidence gathered to date support the hypothesis that the outbreak originated from a strain that was already adapted to humans, as proposed recently [24]. The success of highly virulent human pathogens is easily explained when the human is an accidental host: the pathogen is adapted to its primary host and survives in it, and the accidental high virulence in the human has no consequence for the fitness of the strain in its natural host. However, if the presence of a human reservoir is confirmed, it raises an interesting evolutionary paradox: how could a highly virulent strain subsist in its primary host? One hypothesis would be that the expression of the Shiga toxin confers an evolutionary advantage to its carrier strain. For example, it has been shown that Shiga toxin-carrying enteroaggregative strains have an increased survival in human macrophages [25]. Another hypothesis would be that different human reservoirs might have different sensitivity to VT2-positive O104:H4 strains, and that some human populations might be more resistant than others to the Shiga toxin and would develop less severe symptoms.

Obviously, Shiga-toxin producing *E. coli* is circulating in the human population since such strains have by now been identified in many different countries. The 55989 strain, which has never been typed but which might be of serotype O104:H4 according to unpublished *in silico* serotyping, was isolated in the Central African Republic and 5 strains of VT2-negative O104:H4 *E. coli* were identified in South Africa (2004-2011) [26]. The strain described here, E112/10 was isolated from a patient who had travelled to Tunisia, and out of the three other travel-related cases of patients infected with O104 strains for which the country of travel was identified, one had travelled to Turkey in 2009 and another to Egypt in 2010 [27].

If the Tunisian 2010 isolate had been sequenced immediately upon its isolation, i.e. a year prior to the outbreak, would we have been able to foresee and stop the outbreak before it occurred? The answer is probably not, but hygienic precautions might have been taken to prevent further spread of these and other *E. coli* strains. *E. coli* is our most well studied bacterial model system and a more extensive monitoring of the global *E. coli* population by whole-genome sequencing could help identify both genomic and epidemiological factors associated with increased risks for accidental infectious disease outbreaks.

## Materials and Methods

### Cultivation and DNA Isolation of Bacterial Strains

All strains sequenced in this study were cultivated in Luria Bertani broth over night and DNA was isolated using DNeasy blood and tissue kit from Qiagen. The lysis step (including proteinase K and RNase treatment) was repeated once. The quality of the DNA was analyzed on an agarose gel stained with ethidium bromide and the concentration and purity of the DNA was analyzed using a Nanodrop (Saveen Werner).

### Publicly Available Sequences

Unless otherwise stated, published sequences were retrieved from NCBI (Tables S1 and S2). The genome sequence of the chromosome and plasmids of *E. coli* O104:H4 str. TY-2482 [6] was retrieved from the BGI website (http://climb.genomics.cn/Ecoli_TY-2482). To place the origin of replication at the beginning of the sequence, the genome was rotated and the new start of the sequence placed at position 4033285 in the original assembly. The reads from 7 IonTorrent runs and the Illumina single reads were also retrieved from the same website. IonTorrent reads for strain 01-09591 [8] were retrieved from the Ion Community website (http://lifetech-it.hosted.jivesoftware.com/docs/DOC-1835) and the assembled contigs from NCBI (AFPS00000000.1). Reads and alignments for strains 17-2, JM221, C1010-00, C35-10, C682-09, C734-09, C754-09, C760-09, C777-09 and C227-11 [9] were downloaded from the PacBio Developers Network website (http://www.pacbiodevnet.com/Share/Datasets/E-coli-Outbreak). Sequence and annotation for *E. coli* 55989 were retrieved from the ColiScope database [7]. Gene annotations for the 20 other *E. coli* sequences cited in the same publication were also downloaded from the same source. Reads and assembly for outbreak strains E92/11, and reads from outbreak strains E83/11, E84/11, E90/11, E94/11, E101/11, E103/11, E107/11 are available from NCBI’s Sequence Read Archive [11].

### Genome Sequencing, Assembly and Annotation

The genomes of the 3 strains sequenced in this study were sequenced on a single lane of an Illumina HiSeq2000 run (Illumina, San Diego, CA, USA). It yielded between 8.4 and 17 million 100-bp, short-paired end reads (1.6–3.4 Gb) for each strain. Pyrosequencing of genomic DNA E112/10 was performed on a half plate of 454 Titanium (Roche/454 Life Sciences, Branford, CT, USA), which yielded 614189 reads. Raw reads for all strains sequenced in this study have been deposited at the Sequence Read Archive from the NCBI under study SRP008458.1.

Reads for E112/10 were assembled *de novo* using Newbler 2.5.3 (Roche/454 Life Sciences, Branford, CT, USA), using default settings. It resulted in 217 contigs larger than 300 bp, with an average coverage 36X for 454 reads. Contigs shorter than 300 bp were discarded. The reads from strains C43/90 and C48/93 were assembled *de novo* using Velvet 1.1.05 [28], using a kmer length of 93, determined empirically as the one yielding the longest contigs and the fewest misassemblies.

Contigs from *de novo* assemblies were oriented and ordered by mapping them to the rotated sequence of *E. coli* TY-2482 and its plasmids [6], and to the plasmid pO26-Vir from *E. coli* O26:H11 strain H30 (accession number NC_012487.1) using nucmer of the package MUMmer 3.0 [29]. The best possible tiling was obtained with show-tiling, considering a circular reference, no maximal gap size, a minimum contig length of 1, a minimum identity of 40% and a minimum contig coverage of 30%. Contigs were concatenated following this order, adding unmapped contigs at the end of the assembly.

These concatenated genomes and the one of TY2482 were annotated using a modified version of DIYA [30]. Coding sequences (CDS) were searched for with prodigal 2.0 [31], rRNAs with RNAmmer 1.2 [32], and tRNAs with tRNAscan-SE 1.23 [33]. CDS annotations were based on blastp 2.2.24 [34] searches to a curated collection of *E. coli*-related genomes (ColiScope, [7]), keeping the 10 best blast hits, and the first one as product and gene name. Assembled sequences have been deposited in Genbank (E92/11, AHAU00000000; E112/10, AHAV00000000; C43/90, AHAW00000000; C48/93, AHAX00000000).

### Genome Mapping and SNP Calling

All reads for all strains except those sequenced by PacBio technology [9] were mapped to the complete genome of 2011C-3493 and its plasmids using bwa [35]. IonTorrent and 454 reads were aligned with Burrows-Wheeler/Smith-Waterman alignments (bwasw, [36]) and Illumina reads with Burrows-Wheeler transform (aln/samse, [35]). Whenever applicable, alignments for reads from different technologies were merged with samtools [37]. The remaining of the analysis was done with GATK [38]. Resulting alignments were inspected for regions with bad quality alignments, and realigned if appropriate. Exact duplicated reads, which are artifacts arising from a PCR step occurring during DNA library preparation, were then removed. SNPs were called with UnifiedGenotyper, using a ploidy of 1. To be validated, SNPs had to (i) be covered by reads mapped on both strands, with little strand bias (SB<−1), (ii) be high-quality (QUAL > = 100), (iii) be outside of a homopolymeric region of 3 consecutive identical nucleotides (HRun < = 3).

For genomes sequenced with PacBio and for which alignments in SAM format were available [9] without qualities, the quality from the filtered reads was incorporated in the alignments with ad-hoc scripts. Several SNP-calling programs were evaluated and freebayes 0.8.7 (https://github.com/ekg/freebayes; Erik Garrison, Boston College, Chestnut Hill, MA, USA) was estimated to yield the best results, using a ploidy of 1, a minimum mapping quality of 5, a minimum base quality of 3 and considering only the 2 best alleles.

SNP differences between assembled genomes were identified with the dnadiff and show-snps tools of the MUMmer 3.0 package [29].

Repetitive regions in the reference genome were identified by aligning the whole genome sequence of 2011C-3493 to itself using blastn 2.2.24 [34]. Regions longer than 100 bp that aligned to other regions with >95% identity were deemed as repetitive and SNPs located within them were discarded.

Genome comparisons were performed using blastn 2.2.24 [34], and visualized using ACT [39]. Gene maps were drawn with genoPlotR [40].

### Phylogenetic Analysis

A method similar to the one used by Pandya et al. [41] was used. All complete genomes of *Escherichia coli* and *Shigella* spp. available in Genbank and contigs from the strains 101-1, 04-8351, 09-7901 and NIPH11060424 (Table S1) were aligned to 2011C-3493 with dnadiff from MUMmer 3.0 package [29], and regions that were shared between all strains were identified. These regions were filtered and only the positions for which a SNP in any sequence was present were kept, yielding an alignment of 120,476 positions. The sequences for which only a mapping assembly exists were derived from the alignments to 2011C-3493 and the result of the SNP calling. A maximum-likelihood phylogeny was calculated with RAxML 7.2.7 [42] using these artificial aligned sequences as an input, and a GTR+G model. The tree was rooted according to [7]. The same method was used for the phylogeny of the historical O104:H4 strains, yielding an alignment of 1828 positions.

### PAIs and VT2 Prophage Phylogenies

An online blastn search to the nt database was performed with the sequences of the VT2 prophage and the identified pathogenicity islands PAI 1 and 2. From the coverage of the blast results, the VT2 prophage, PAI 1 and 2 were split in 2, 4 and 2 fragments, respectively (Figures 5, 6 and 7). Each fragment was subsequently used as a query to blastn against the nt database available at NCBI. A representative subset of the best blast hits was selected and the corresponding sequences were downloaded (Table S2), aligned with mafft [43]. Columns of the alignment that contained more than 50% of gaps were removed. A maximum likelihood phylogeny was then calculated with RAxML 7.2.7 [42] using the resulting alignment as input and GTR+G as a model.

## Supporting Information

Figure S1 Phylogeny of *E. coli* and *Salmonella* genomes. Maximum-likelihood phylogenies of all *E. coli* and *Salmonella* strains for which genome data is available, including C35-10, JM221 and 17_2, which were sequenced with PacBio. Font coloring, red: O104:H4 strains; blue: EAEC of non-O104:H4 serotype; green: EHEC strains.
(PDF)

Table S1 Accession numbers for complete genome sequences or contigs included in the whole-genome phylogeny.
(PDF)

Table S2 Accession numbers for sequences included in the pathogenicity islands and phage phylogenies.
(PDF)

Dataset S1 (XLS)
