# Supplementary figures and images for: Adaptive Mutations and Replacements of Virulence Traits in the Escherichia coli O104:H4 Outbreak Population
Source: PLoS One. 2013 May 10;8(5):e63027. doi: 10.1371/journal.pone.0063027 (PMC3651199; doi:10.1371/journal.pone.0063027)

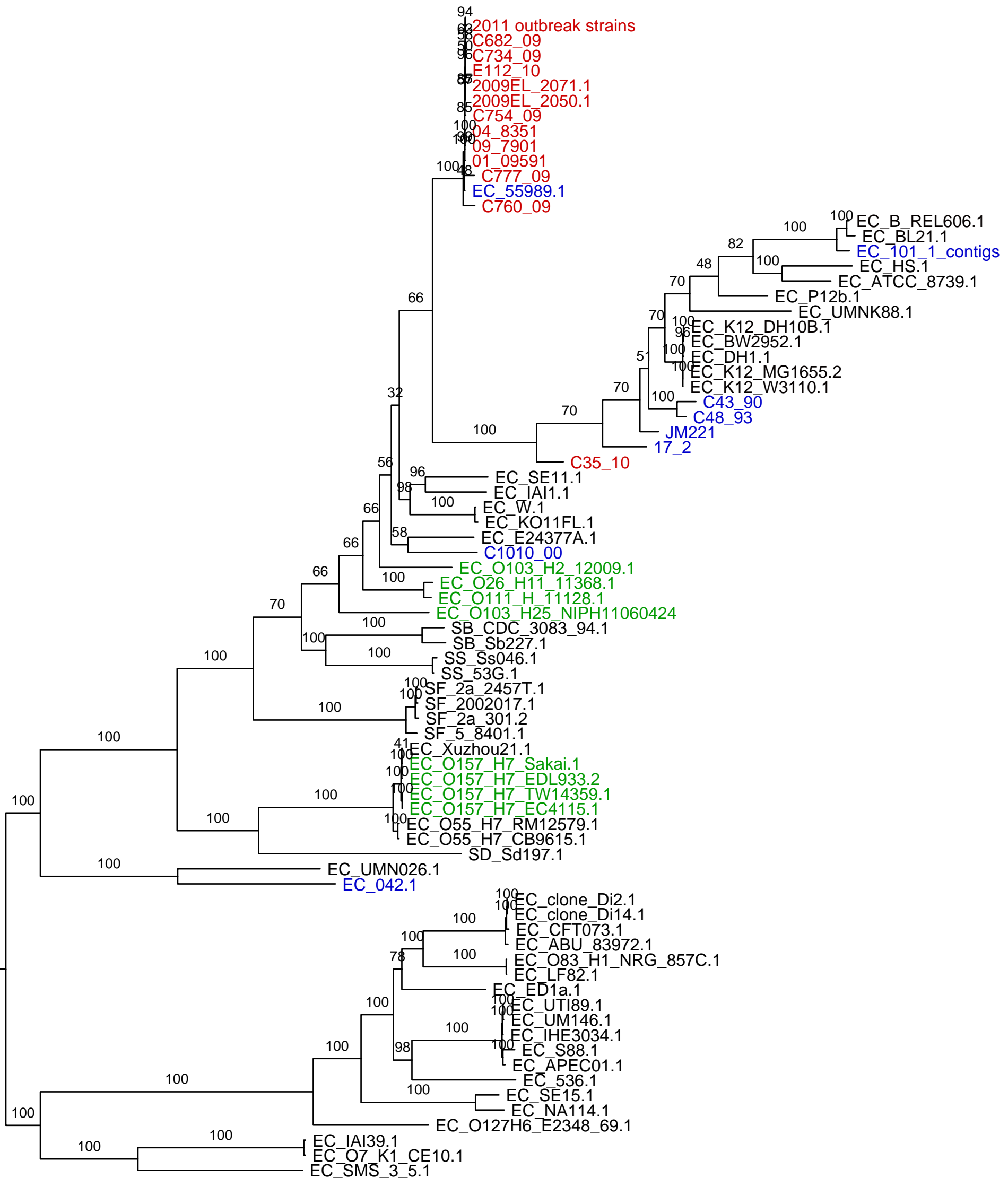

0.04

Supplement: Figure S1 — Phylogeny of E. coli and Salmonella genomes. Maximum-likelihood phylogenies of all E. coli and Salmonella strains for which genome data is available, including C35-10, JM221 and 17_2, which were sequenced with PacBio. Font coloring, red: O104:H4 strains; blue: EAEC of non-O104:H4 serotype; green: EHEC strains. (PDF) [file pone.0063027.s001.pdf]
