# Supplementary material for: Adaptive Mutations and Replacements of Virulence Traits in the Escherichia coli O104:H4 Outbreak Population
Source: PLoS One. 2013 May 10;8(5):e63027. doi: 10.1371/journal.pone.0063027 (PMC3651199; doi:10.1371/journal.pone.0063027)
Supplement: Table S1 — Accession numbers for complete genome sequences or contigs included in the whole-genome phylogeny. (PDF) [file pone.0063027.s002.pdf]

**Table S1:** Accession numbers for complete genome sequences or contigs included in the whole-genome phylogeny

| Name | Accession number |
|---|---|
| EC_042 | NC_017626 |
| EC_536 | NC_008253 |
| EC_55989 | NC_011748 |
| EC_ABU_83972 | NC_017631 |
| EC_APEC01 | NC_008563 |
| EC_ATCC_8739 | NC_010468 |
| EC_BL21 | NC_012947 |
| EC_B_REL606 | NC_012967 |
| EC_BW2952 | NC_012759 |
| EC_CFT073 | NC_004431 |
| EC_E24377A | NC_009801 |
| EC_clone_Di14 | NC_017652 |
| EC_clone_Di2 | NC_017651 |
| EC_DH1 | NC_017625 |
| EC_ED1a | NC_011745 |
| EC_HS | NC_009800 |
| EC_IAI1 | NC_011741 |
| EC_IAI39 | NC_011750 |
| EC_IHE3034 | NC_017628 |
| EC_K12_DH10B | NC_010473 |
| EC_K12_MG1655 | NC_000913 |
| EC_K12_W3110 | NC_007779 |
| EC_KO11FL | NC_016902 |
| EC_LF82 | NC_011993 |
| EC_NA114 | NC_017644 |
| EC_O103_H2_12009 | NC_013353 |
| EC_O111_H_11128 | NC_013364 |
| EC_O127H6_E2348_69 | NC_011601 |
| EC_O157_H7_EC4115 | NC_011353 |
| EC_O157_H7_EDL933 | NC_002655 |
| EC_O157_H7_Sakai | NC_002695 |
| EC_O157_H7_TW14359 | NC_013008 |
| EC_O26_H11_11368 | NC_013361 |
| EC_O55_H7_CB9615 | NC_013941 |
| EC_O55_H7_RM12579 | NC_017656 |
| EC_O7_K1_CE10 | NC_017646 |
| EC_O83_H1_NRG_857C | NC_017634 |
| EC_P12b | NC_017663 |
| EC_S88 | NC_011742 |
| EC_SE11 | NC_011415 |
| EC_SE15 | NC_013654 |
| EC_SMS_3_5 | NC_010498 |
| EC_UM146 | NC_017632 |
| EC_UMN026 | NC_011751 |
| EC_UMNK88 | NC_017641 |
| EC_UTI89 | NC_007946 |

| **Name** | **Accession number** |
|---|---|
| EC_W | NC_017635 |
| EC_Xuzhou21 | NC_017906 |
| SB_CDC_3083_94 | NC_010658 |
| SB_Sb227 | NC_007613 |
| SD_Sd197 | NC_007606 |
| SF_2002017 | NC_017328 |
| SF_2a_2457T | NC_004741 |
| SF_2a_301 | NC_004337 |
| SF_5_8401 | NC_008258 |
| SS_53G | NC_016822 |
| SS_Ss046 | NC_007384 |
| EC_101_1_contigs | NZ_AAMK020000* |
| 04_8351 | AFRL01 |
| 09_7901 | AFRK01 |
| EC_O103_H25_NIPH11060424 | AGSG01 |
| 2011C_3493 | NC_018658 |
| 2009EL_2050 | NC_018650 |
| 2009EL_2071 | NC_018661 |
