# Supplementary material for: Adaptive Mutations and Replacements of Virulence Traits in the Escherichia coli O104:H4 Outbreak Population
Source: PLoS One. 2013 May 10;8(5):e63027. doi: 10.1371/journal.pone.0063027 (PMC3651199; doi:10.1371/journal.pone.0063027)
Supplement: Table S2 — Accession numbers for sequences included in the pathogenicity islands and phage phylogenies. (PDF) [file pone.0063027.s003.pdf]

Table S2: Accession numbers for sequences included in the pathogenicity islands and phage phylogenies

**Name**	**Accession number**
CS_BAA_894	CP000783
KP_pK2044	AP006726
KP_pK29	EF382672
E_cloacae_pEC_IMPQ	EU855788
EC_pAPEC_01_R	DQ517526
EC_pNT3B	AJ888883
E_cloacae_pEC_IMP	EU855787
SM_R478	BX664015
EC_UMNK88	CP002729
KP_str342_pKP187	CP000965
E_cloacae_13047_pECL_A	CP001919
KP_pLVPK	AY378100
EC_ABU_83972	CP001671
EC_mic_op_str_CA46	AJ515251
EC_042	FN554766
EC_mic_op_str_CA58	AJ515252
EC_mic_op_H47	AJ009631
EC_DH1	CP001637
EC_O83_H1_str_857C_p083_CORR	CP001856
KP_pKP048	FJ628167
EC_O111_H_11128_pO111_1	AP010961
SE_strCT18_pHCM1	AL513383
ST_pU302L	AY333434
EC_O26_H_06877_pO26_CRL	GQ259888
EC_APEC03_pAPEC_0103_ColBM	CP001232
SE_ParatA_IncH1	AM412236
EC_SE15	AP009378
SF_2a_SRL_path_island	AF326777
Entbact_phage_VT2_Sakai	AP000363
Entbact_phage_VT2phi_272	HQ424691
NC_009801	CP000800
NC_013353	AP010958
NC_013008	CP001368
NC_002695	BA000007
NC_013364	AP010960
NC_011745	CU928162
NC_013361	AP010953
NC_011748	CU928145
NC_008253	CP000247
NC_002655	AE005174
NC_011353	CP001164
NC_004741	AE014073
NC_000913	U00096
NC_010473	CP000948
NC_012759	CP001396
EC_536	NC_008253
EC_55989	NC_011748

Name	Accession number
EC_APEC01	NC_008563
EC_ATCC_8739	NC_010468
EC_BL21	NC_012947
EC_B_REL606	NC_012967
EC_BW2952	NC_012759
EC_CFT073	NC_004431
EC_E24377A	NC_009801
EC_ED1a	NC_011745
EC_HS	NC_009800
EC_IAI1	NC_011741
EC_IAI39	NC_011750
EC_K12_DH10B	NC_010473
EC_K12_MG1655	NC_000913
EC_K12_W3110	AC_000091
EC_O103_H2_12009	NC_013353
EC_O111_H_11128	NC_013364
EC_O127H6_E2348_69	NC_011601
EC_O157_H7_EC4115	NC_011353
EC_O157_H7_EDL933	NC_002655
EC_O157_H7_Sakai	NC_002695
EC_O157_H7_TW14359	NC_013008
EC_O26_H11_11368	NC_013361
EC_O55_H7_CB9615	NC_013941
EC_S88	NC_011742
EC_SE11	NC_011415
EC_SMS_3_5	NC_010498
EC_UMN026	NC_011751
EC_UTI89	NC_007946
SB_CDC_3083_94	NC_010658
SB_Sb227	NC_007613
SD_Sd197	NC_007606
SF_2a_2457T	NC_004741
SF_2a_301	NC_004337
SF_5_8401	NC_008258
SS_Ss046	NC_007384
EC_K12_W3110	AP009048
Stx1_conv_phage	AP005153
Stx2_conv_phage_I	AP004402
Stx2_conv_phage_II	AP005154
Entbact_phage_Min27	EU311208
SD_Sd197	CP000034
EC_O26_H11_11368	AP010953
phage_TL2011c	JQ011318
Stx2_conv_phage_86	AB255436
2011C3493	NC_018658
2009EL2050	NC_018650
2009EL2071	NC_018661
Stx2_convph_vB_EcoP_24B	HM208303
